# Changes in dominant groups of the gut microbiota do not explain cereal-fiber induced improvement of whole-body insulin sensitivity

**DOI:** 10.1186/1743-7075-8-90

**Published:** 2011-12-17

**Authors:** Martin O Weickert, Ayman M Arafat, Michael Blaut, Carl Alpert, Natalie Becker, Verena Leupelt, Natalia Rudovich, Matthias Möhlig, Andreas FH Pfeiffer

**Affiliations:** 1 Department of Clinical Nutrition, German Institute of Human Nutrition, Potsdam-Rehbruecke, Germany; 2 Department of Endocrinology, Diabetes and Nutrition, Charité-University-Medicine Berlin, Germany; 3 Warwickshire Institute for the Study of Diabetes, Endocrinology and Metabolism, University Hospitals Coventry and Warwickshire NHS Trust, Coventry, UK; 4 Clinical Sciences Research Institute, Warwick Medical School, University of Warwick, Coventry, UK; 5 Department of Gastrointestinal Microbiology, German Institute of Human Nutrition, Potsdam-Rehbruecke, Germany

**Keywords:** cereal fiber, whole-body insulin sensitivity, gut microbiota, short chain fatty acids (SCFA), colonic fermentation

## Abstract

**Background:**

Diets high in cereal-fiber (HCF) have been shown to improve whole-body insulin sensitivity. In search for potential mechanisms we hypothesized that a supplemented HCF-diet influences the composition of the human gut microbiota and/or biomarkers of colonic carbohydrate fermentation.

**Methods:**

We performed a randomized controlled 18-week intervention in group-matched overweight participants. Fecal samples of 69 participants receiving isoenergetic HCF (cereal-fiber 43 g/day), or control (cereal-fiber 14 g/day), or high-protein (HP, 28% of energy-intake, cereal-fiber 14 g/day), or moderately high cereal fiber/protein diets (MIX; protein 23% of energy-intake, cereal-fiber 26 g/day) with comparable fat contents were investigated for diet-induced changes of dominant groups of the gut microbiota, and of fecal short-chain fatty-acids (SCFA) including several of their proposed targets, after 0, 6, and 18-weeks of dietary intervention. *In vitro *fermentation of the cereal fiber extracts as used in the HCF and MIX diets was analyzed using gas chromatography. Diet-induced effects on whole-body insulin-sensitivity were measured using euglycaemic-hyperinsulinemic clamps and re-calculated in the here investigated subset of n = 69 participants that provided sufficient fecal samples on all study days.

**Results:**

Gut microbiota groups and biomarkers of colonic fermentation were comparable between groups at baseline (week 0). No diet-induced differences were detected between groups during this isoenergetic intervention, neither in the full model nor in uncorrected subgroup-analyses. The cereal-fiber extract as used for preparation of the supplements in the HCF and MIX groups did not support *in vitro *fermentation. Fecal acetate, propionate, and butyrate concentrations remained unchanged, as well as potential targets of increased SCFA, whereas valerate increased after 6-weeks in the HP-group only (p = 0.037). Insulin-sensitivity significantly increased in the HCF-group from week-6 (baseline M-value 3.8 ± 0.4 vs 4.3 ± 0.4 mg·kg^-1^·min^-1^, p = 0.015; full model 0-18-weeks, treatment-x-time interaction, p = 0.046).

**Conclusions:**

Changes in the composition of the gut microbiota and/or markers of colonic carbohydrate fermentation did not contribute explaining the observed early onset and significant improvement of whole-body insulin sensitivity with the here investigated HCF-diet.

**Trial registration:**

This trial was registered at http://www.clinicaltrials.gov as NCT00579657

## Background

It is generally assumed that the beneficial effects of insoluble cereal fiber arise from chronic intake and its subsequent fermentation in the colon [1-4]. In prospective cohort studies cereal fiber intake is associated with improved insulin sensitivity [5,6] and reduced risk for developing Type 2 Diabetes, after correction for confounders [7,8]. In randomized controlled interventions several [9-12], but not all [13] previous studies show improved whole-body insulin sensitivity with HCF diets, with the effects likely being dose dependent [12]. However, most cereal fibers are fermented only moderately [14]. Since reduced risk of diabetes is most consistently observed with a high intake of cereal fibers, as opposed to more readily fermentable sources of fiber such as fruit and vegetables [7,8], it is not clear whether changes in the gut microbiota and/or colonic fermentation are indeed key factors involved in conveying improved insulin sensitivity with a HCF intake.

In rodent models highly fermentable fibers such as oligofructose and resistant starch have been shown to lead to short chain fatty acid (SCFA)-induced up-regulation of pro-glucagon mRNA, resulting in increased glucagon like peptide-1 (GLP-1) and other factors that are assumed to have beneficial metabolic effects [15,16]. However, results in human studies are controversial [17-20]: as an example, exposure to highly fermentable resistant starch did not result in any detectable changes in circulating GLP-1 concentrations, despite significant improvement of insulin sensitivity [17,20]. Furthermore, it has been shown recently that measurable changes of circulating GLP-1 concentrations due to a high intake of cereal fiber may need as long as 9 months of continued ingestion in humans [1].

No previous studies in humans are available that performed parallel investigation of diet induced changes in dominant groups of the human gut microbiota, markers of colonic carbohydrate fermentation, and state of the art measurement of insulin sensitivity. We have recently shown that the here used isoenergetic HCF diet significantly improves whole-body insulin sensitivity by 16% from 6-weeks of dietary intervention [12]. We here investigated whether improved insulin sensitivity in response to the HCF diet is associated with changes in markers of colonic carbohydrate fermentation and/or in the composition of dominant groups of the human gut microbiota, concepts that could further contribute explaining the consistently observed beneficial metabolic effects of a HCF intake [7,8,14].

## Methods

### Study design

This study had been approved by the Ethics Committee of the University of Potsdam (BMBF FKZ 0313826). All participants had given their written informed consent. Primary outcome measures were diet-induced changes in whole-body and hepatic insulin sensitivity. Factors that might contribute to explain altered insulin sensitivity were secondary outcomes that included the measurement of markers of colonic carbohydrate fermentation and dominant groups of the human gut microbiota (Clinicaltrials.gov NCT00579657).

Effects of the diets on the primary outcomes and details of the dietary intervention have been published [12]. In brief, 111 overweight adults with features of the metabolic syndrome were randomly assigned to one of four two-phased, 18-weeks isoenergetic diets, by group-matching; the percentages of energy derived from protein and carbohydrates, and intake of cereal fiber per day were 17%/52%/14 g (control); 17%/52%/43g (HCF); 28%/43%/13g (HP); and 23%/44%/26g (MIX) after 6-weeks, and 17%/51%/15g (control); 17%/51%/41g (HCF); 26%/45%/14g (HP); and 22%/46%/26g (MIX) after 18-weeks, according to 3-day food protocols. Fat-intake was aimed to be 30% of energy in all groups. Adherence was supported by intense dietary advice and the provision of tailored dietary supplements twice daily in all groups.

Out of the n = 111 participants that were randomised to their respective diets, n = 84 participants fulfilled the all inclusion criteria after 18-weeks of dietary intervention (i.e. no relevant changes in body weight during this isoenergetic intervention, no intake of drugs known to influence insulin sensitivity, such as cortisone and antibiotics, and no major problems with dietary adherance), as detailed elsewhere [12]. Analyses in the present study were focused on potential changes of markers of colonic carbohydrate fermentation and dominant groups of the human gut microbiota in the HCF group, which could contribute explaining the observed HCF diet-induced improvement of insulin sensitivity [12]. Sufficient fecal samples (correctly sampled fecal material on all 3 study days) were available in n = 69 of these participants. All analyses were performed in this subset, after 0, 6 and 18 weeks of dietary intervention.

### Subjects

Details have been published [12]. In brief: for eligibility participants had to be 24 - 70 years of age and to be overweight (body-mass-index (BMI) >25 kg/m² (n = 45) or obese (BMI > 30 kg/m², n = 66), with a waist circumference > 80 cm in females and > 94 cm in males, and show at least one more feature of the metabolic syndrome according to IDF criteria [21]. Characteristic of glucose metabolism (normal fasting glucose, impaired fasting glucose and/or impaired glucose tolerance, according to oral glucose tolerance tests) were comparable between groups. Major exclusion criteria were pregnancy, diabetes, diseases of heart, liver, or kidneys, allergies including food allergies, and metal implants. Further exclusion criteria were relevant changes in body weight and/or physical activity during the study, the intake of drugs known to affect insulin sensitivity which included the intake of antibiotics, and relevant problems with dietary adherence [12].

### Measurement of changes in dominant groups of the gut microbiota

Fecal collection, fluorescence in situ hybridization (FISH) and bacterial enumeration by flow cytometry were performed essentially as described [22]. In brief, freshly voided fecal samples were transported in air-tight plastic boxes under anaerobic conditions (AnaeroGen, Oxoid) and stored at 4°C. They were processed within 3 hours after defecation by paraformaldehyde fixation as described previously [22]. After fixation bacterial cells were washed once with PBS, then resuspended in PBS and one volume of ethanol was added. Samples were stored at -80°C until analysis. For in situ hybridization, fixed bacterial cells were washed once in 10× PBS and once in Tris-EDTA buffer (100 mM Tris-HCl [pH 8.0], 50 mM EDTA). The following lysozyme treatment for permeabilization and the hybridization with the respective group-specific (Cy5 labelled) and all bacteria detecting EUB 338 (FITC labelled) probes (additional file 1, Table S1) and stringency washing procedures were described by Mueller et al [22]. Flow cytometry data acquisition was performed with a FACS Calibur flow cytometer (Becton Dickinson) as described previously [23] using the settings of Mueller et al [22].

Cells belonging to a bacterial group or species were enumerated with a group or species-specific probe as the relative proportion of the cells detected with the EUB 338 probe, which detects all bacteria. This proportion was corrected by subtracting the background fluorescence obtained with the negative control probe NONEUB 338.

Changes in gut microbiota were measured at 0, 6, and 18 weeks, for the following groups with the respective oligonucleotide probe given in brackets: *Clostridium coccoides *- *Eubacterium rectale *cluster (Erec482), *Roseburia *genus (Rrec584), *Bacteroides-Prevotella *(Bac303), *Clostridium leptum *cluster (Clep866), *Atopobium *cluster (Ato291), *Bifidobacterium *group (Bif164), and clostridial cluster IX (Prop853). *Lactobacillus-Streptococcus *group (Lab158) and *Enterobacteria *(Enter1432) were under the detection limit. Total bacteria were detected with the EUB338 probe. Details about the here used probes and primer sequences are given in the additional file 1, Table S1. The selected oligonucleotide probes target major bacterial population groups. *Roseburia, E. rectale *and *Faecalibacterium *groups are major butyrate producers, while *E. rectale*, *Roseburia, Bacteroides-Prevotella *and *Bifidobacterium *respond to the supply of peptides [24-26]. Intestinal *Roseburia *decreases significantly in human subjects on a low carbohydrate diet [27].

### Measurement of in vitro fermentation of the cereal fiber extract as used in the HCF and MIX diets

Fecal samples were collected from a healthy volunteer (age 25) who had no previous history of gastrointestinal disorders and had not undergone antibiotic therapy within the last six months. A fresh fecal sample was diluted ten-fold (w/v) in pre-reduced phosphate-buffered saline (PBS) (contents per liter: 8.5 g of NaCl, 0.3 g of KH_2_PO_4_, 0.6 g of Na_2_HPO_4_, 0.1 g of peptone, 0.25 g of cysteine) and centrifuged (1 min, 300× g) to remove non-bacterial particles. Before inoculation, the fecal bacteria were washed three times with pre-reduced PBS (5 min, 8000× g). Ten-fold diluted bacterial suspension was transferred to the fermentation vessels (0.2% inoculum). Fermentations were carried out in 250 ml glass vessels containing 125 ml bicarbonate buffered medium (BIC) [contents per liter: 0.35 g of K_2_HPO_4_, 0.23 g of KH_2_PO_4_, 0.5 g of NH_4_Cl, 2.25 g of NaCl, 0.5 g of MgSO_4_*7H_2_0, 0.07 g of CaCl_2_*2 H_2_O, 0.005 g of FeSO_4_*7H_2_O, 0.00015 g of NaSeO_3_, 3.5 mg of tryptically digested peptone from casein, 3.5 mg of yeast extract, 0.5 g of cysteine*HCl, 4.0 g of NaHCO_3 _and 3 ml of trace element solution SL10 [28]. Medium (125 ml) was added to 0.25 g of glucose or the cereal fiber extract (HF101). The pH was adjusted to 7.5, the medium was gassed with N_2_/CO_2 _(80:20 v/v) and autoclaved at 121°C for 15 min. Following autoclaving five-fold concentrated vitamin solution (4 ml/100 ml) was added [29]. Aliquots were taken after 0, 24 and 46 h of incubation and analyzed. The pH was measured directly in the samples by using a MultiCal pH-meter (WTW, Weilheim, Germany).

Measurements of optical density (OD) and in vitro production of short-chain fatty acids (SCFA) were performed as follows: One ml batch culture was centrifuged (5 min, 14000 × g), the pellet was re-suspended in PBS (1 ml) and the resulting OD was determined at 600 nm (DU-640 spectrophotometer, Beckman Instruments Inc., Fullerton, CA). Gas chromatography (GC) of SCFA was performed as described above.

### Measurement of short chain fatty acids (SCFA) in fecal samples

Fresh feces (300 mg) were diluted 5-fold in water and centrifuged at 15000 × g for 5 min. Isobutyric acid (23.6 µl 12 mM, internal standard), 280 µl 0.36 M HClO_4_, and 270 µl 1 M NaOH were added to 200 µl of the supernatant. The mixture was lyophilized, and the residue was re-dissolved in a mixture of 400 µl acetone and 100 µl 5 M formic acid. After centrifugation at 14,000 × g for 5 min at room temperature, 1 µl of the supernatant was injected into the gas chromatograph. Authentic standards were incorporated in all runs.

Acetate (C2), propionate (C3), butyrate (C4), valerate (C5) and isovalerate (iso-C5) were measured with an HP 5890 series II gas chromatograph (Hewlett-Packard, Waldbronn, Germany) equipped with a HP-20 M (Carbowax 20 M) column (30 m × 0.53 mm; film thickness 0,3 µm) and a flame ionization detector. Helium was used as carrier gas at a flow rate of 1 ml/min. The initial column temperature of 75°C for 1 min was subsequently increased at a rate of 20°C/min to 100°C and then to 150°C at a rate of 5°C/min. The final temperature of 150°C was maintained for 3 min. The temperature of the injector and of the detector was 200°C. The split ratio was 1:10.

### Blood tests

Routine laboratory markers were measured by using standard methods in the research laboratories of the German Institute of Human Nutrition. Glucose concentrations were measured in venous blood (ABX Pentra 400; ABX Diagnostics, Montpellier, France), and additionally, for the clamp studies, in arterialized blood samples. Arterialized plasma glucose concentrations were measured immediately by using the glucose oxidase method (Super-GL glucose analyzer; Dr. Müller, Freital, Germany). Plasma plasminogen activator inhibitor-1 was measured by using an enzyme-linked immunosorbent assay (IBL, Hamburg, Germany; intra-assay CV: 4.7%; inter-assay CV: 5%). Serum interleukin-10 (IL-10) was measured by using a highly sensitive immune assay (Quantikine, Wiesbaden, Germany; intra-assay CV: 6.6%; inter-assay CV: 8.1%). C-reactive protein was measured by using turbidimetric immunoprecipitation on an ABX Pentra 400 (ABX Diagnostics; intra-assay CV: 1.6%; inter-assay CV: 4.3%). Ghrelin was measured using an enzyme immunometric assay (SPIbio, Montigny de Bretonneux, France; intra-assay CV: 7.5%; inter-assay CV: 8.2%).

### Euglycaemic hyperinsulinaemic clamps

Euglycaemic hyperinsulinaemic clamps were performed as detailed previously [12].

### Power calculation

This study was powered to detect diet-induced differences in whole-body insulin sensitivity between the HCF and the HP groups [12]. Assuming a dropout rate of 30% we required 26 participants for each treatment. However, the sample size of the here investigated subset of participants who provided sufficient fecal samples on all study days was adequate to show significant diet-induced differences between groups on whole-body insulin sensitivity, both in the full model and in Bonferroni corrected sub-analyses. Therefore, it was reasonable to assume that sample size was sufficient for the detection of factors that may contribute explaining the here observed changes of whole-body insulin sensitivity.

### Statistical analyses

Results are presented as mean ± SEM. Analyses of this proof of principle nutritional intervention were performed according to the study protocol (ClinicalTrials.gov number, NCT00579657), in an attempt to exclude confounding factors with known effects on the main outcome measure (insulin sensitivity) that are likely to obscure diet-induced effects. Participants who reported that they were not able or willing to adhere to their respective diets were also excluded. Details of all inclusion and exclusion criteria have been published [12].

Eighty-four of the participants (controls, n = 22, HCF, n = 18; HP, n = 22; and MIX, n = 22) completed the dietary intervention successfully [12]. Of those 69 subjects provided sufficient fecal samples on all study days (controls, n = 18, HCF, n = 16; HP, n = 19; and MIX, n = 16, Table 1). All here presented analyses were performed in all participants. Diet-induced changes in whole-body insulin sensitivity were re-calculated in this subset and analysed using a mixed-model analysis for repeated measures, including the 4 dietary treatments and 3 time points (weeks 0, 6, and18) into one model. For separate investigation of dietary effects at weeks 0, 6 and 18, respectively, one-factor analysis of variance (ANOVA) with Bonferroni adjustment for post hoc comparisons was performed. Not normally distributed data were analysed using Kruskal-Wallis test with Mann-Whitney U-tests for comparisons in subgroups. In order to detect potentially present smaller differences in markers of colonic fermentation and dominant groups of the gut microbiota between dietary intervention groups, we additionally performed uncorrected subgroup analyses for these parameters even if there was no significant effect in the full model. Longitudinal changes within groups were addressed by two-tailed Students' T-test for paired samples. p < 0.05 was considered significant. Analyses were performed using SPSS version 16 (Chicago, Illinois, USA).

**Table 1:** Baseline characteristics of the participants

Characteristics of participants	Control	HCF	HP	MIX	p value^1^
Gender (males/females)	8/10	5/11	7/12	6/10	0.93
Age - yr	55.3 ± 1.9	54.2 ± 3.2	57.4 ± 1.8	55.3 ± 1.9	0.76
Waist circumference - cm	101.6 ± 2.1	101.4 ± 1.9	101.4 ± 2.6	100.6 ± 3.3	0.99
BMI (kg/m^2^)	31.0 ± 0.8	31.9 ± 0.8	31.4 ± 0.9	31.4 ± 1.0	0.92
					
M-value (mg·kg^-1^·min^-1^)	4.4 ± 0.5	3.8 ± 0.4	4.0 ± 0.4	4.4 ± 0.4	0.67
Fecal SCFA (mM)					
Acetate (C2)	40.2 ± 3.1	38.8 ± 4.5	39.3 ± 4.4	39.8 ± 3.9	0.88
Propionate (C3)	12.4 ± 1.1	13.3 ± 1.4	13.0 ± 1.3	15.1 ± 1.5	0.64
Butyrate (C4)	12.3 ± 1.5	12.8 ± 2.4	13.6 ± 1.9	13.1 ± 1.7	0.98
Valerate (C5)	2.3 ± 0.3	2.0 ± 0.3	2.2 ± 0.2	2.2 ± 0.2	0.66
Sum SCFA^1^ Gut microbiota groups (%)	70.8 ± 5.3	69.5 ± 7.8	71.6 ± 6.7	73.1 ± 6.5	0.90
Bac303	7.1 ± 1.8	5.4 ± 1.4	5.5 ± 1.9	9.4 ± 3.0	0.72
Erec482	26.1 ± 2.7	24.3 ± 3.4	23.2 ± 2.9	23.0 ± 2.7	0.76
Rrec584	10.0 ± 2.2	10.6 ± 2.4	8.6 ± 1.8	6.9 ± 1.4	0.78
Clep866	11.4 ± 1.2	13.6 ± 1.7	14.1 ± 2.0	10.7 ± 1.7	0.66
Ato291	2.4 ± 0.6	2.8 ± 1.0	3.5 ± 0.7	3.8 ± 0.7	0.25
Bif164	4.0 ± 1.0	2.2 ± 0.8	2.7 ± 0.7	2.7 ± 1.0	0.46
Prop853	4.9 ± 1.2	7.9 ± 1.3	9.5 ± 1.8	6.7 ± 1.6	0.13
Fecal dry mass (%)	26.0 ± 1.8	28.2 ± 2.7	26.3 ± 1.8	26.4 ± 1.8	0.98

All values are mean ± SEM. HCF, diet high in cereal fiber; HP, diet high in protein; MIX, diet moderately high in both cereal and protein. M value, insulin mediated glucose uptake as a measurement for whole body insulin sensitivity. SCFA, fecal short chain fatty acids.

Bac303, *Bacteroides-Prevotella*; Erec482, *Clostridium coccoides *- *Eubacterium rectale *cluster; Rrec584, *Roseburia *genus, Clep866, *Clostridium leptum *cluster; Ato291, *Atopobium *cluster; Bif164, *Bifidobacterium *group; Prop853, *clostridial cluster IX*. Total bacteria were detected with the EUB338 probe.

^1 ^P values refer to the respective treatment × time interaction in the full model

^2^Sum SCFA includes the measurement of fecal iso-acids

## Results

### Dietary effects on dominant groups of the gut microbiota

Fecal samples collected after 0, 6 and 18 weeks of dietary intervention were analyzed in duplicate for diet-induced changes of dominant groups of the gut microbiota using FISH coupled with flow cytometry. The distribution of the targeted gut microbial groups was comparable between the participants at baseline (Table 1). In the full model there were no significant diet-induced changes in any of the here investigated dominant groups of the human gut microbiome, p > 0.23 for all measured gut microbiota groups after 6 weeks, and p > 0.58 for all groups after 18 weeks (Table 2). There were also no diet-induced significant differences between groups after normalisation for the baseline (p > 0.17 for all measured gut microbiota groups after 6 weeks; and p > 0.18 for all groups after 18 weeks).

**Table 2:** Dominant groups of the gut microbiota, according to diet, after 6 and 18 weeks.

		6 weeks					18 weeks			
				
	control	HCF	HP	MIX	p value	control	HCF	HP	MIX	p value^1^
Bac303 (%)	9.9 ± 3.2	6.4 ± 1.7	5.4 ± 1.7	7.2 ± 1.5	0.65	7.1 ± 1.6	8.0 ± 2.0	7.9 ± 1.9	7.4 ± 2.4	0.88
Erec482 (%)	24.2 ± 3.2	27.9 ± 3.9	21.4 ± 3.1	25.2 ± 2.6	0.53	23.2 ± 2.5	24.2 ± 3.2	22.7 ± 1.6	23.5 ± 3.1	0.99
Rrec584 (%)	9.7 ± 1.9	12.6 ± 2.5	6.5 ± 1.3	8.0 ± 1.5	0.23	7.6 ± 1.5	8.2 ± 1.8	6.0 ± 1.2	8.4 ± 2.2	0.86
Clep866(%)	11.4 ± 1.4	12.5 ± 1.6	13.2 ± 1.4	12.3 ± 1.7	0.64	12.3 ± 1.5	14.5 ± 1.5	15.4 ± 1.7	13.2 ± 1.5	0.58
Ato291 (%)	2.9 ± 0.7	3.8 ± 1.1	5.2 ± 1.1	4.6 ± 1.0	0.25	3.2 ± 0.6	3.8 ± 0.7	4.9 ± 0.9	3.9 ± 0.4	0.67
Bif164 (%)	4.6 ± 1.1	4.0 ± 1.1	2.3 ± 0.8	2.1 ± 0.5	0.27	3.0 ± 0.9	2.9 ± 0.8	2.7 ± 0.8	2.8 ± 0.6	0.92
Prop853 (%)	5.3 ± 1.2	6.9 ± 1.6	6.2 ± 1.0	5.7 ± 1.2	0.83	5.9 ± 1.1	7.0 ± 1.9	6.7 ± 1.5	5.3 ± 1.3	0.94

All values are mean ± SEM. Baseline values are shown in Table 1. HCF, diet high in cereal fiber; HP, diet high in protein; MIX, diet moderately high in both cereal and protein. ^1^P values refer to the respective treatment × time interaction in the full model. Bac303, *Bacteroides-Prevotella*; Erec482, *Clostridium coccoides *- *Eubacterium rectale *cluster; Rrec584, *Roseburia *genus, Clep866, *Clostridium leptum *cluster; Ato291, *Atopobium *cluster; Bif164, *Bifidobacterium *group; Prop853, *clostridial cluster IX*. Total bacteria were detected with the EUB338 probe.

Although no significant effects of the four diets were detected in the full model, we additionally performed subgroup analyses in order to detect potentially present smaller differences between dietary groups. Again, no significant differences were detected between groups both after 6 and 18 weeks. *Roseburia *tended to be increased in the HCF as compared to the HP group after 6 weeks of dietary intervention (12.6 ± 2.5 vs 6.5 ± 1.3%, p = 0.068), but not after 18 weeks (p = 0.37). No further trends were observed between dietary groups (p > 0.19 for all other measured gut microbiota groups). Re-analysing all the microbiota data after normalizing for baseline value levels (week 0) again did not show any significant diet-induced effects.

When investigating longitudinal diet-induced changes within groups, the following changes were detected: controls, no significant changes (p > 0.21 for all gut microbiota groups after 6 weeks, and p > 0.21 after 18 weeks; *Bifidobacterium *tended to decrease between week 6 and week 18 (p = 0.079); HF, no significant changes (p > 0.17 for all gut microbiota groups after 6 weeks; p > 0.15 for all groups after 18 weeks); HP: Members of *clostridial cluster IX *decreased after 6-weeks of HP diet (p = 0.017), but there was no significant change between week 0 and week 18 (p = 0.11; Table 2). *Atopobium *tended to increase after 6 weeks in the HP group (p = 0.095) and was significantly increased versus the baseline after 18 weeks (p = 0.024, Table 2). No longitudinal changes in gut microbiota groups were observed with the MIX diet.

### In vitro fermentation of the cereal fiber extract

**Figure 1:**
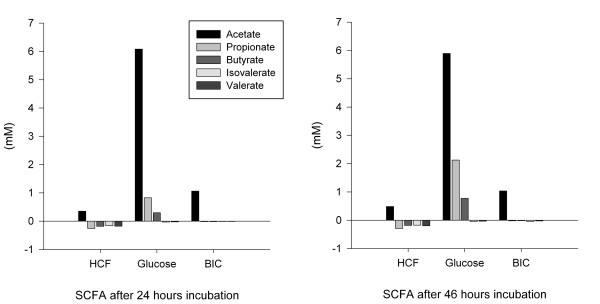
**In vitro fermentation of the cereal fiber extract**. The figure shows in vitro fermentation of glucose in comparison to the cereal fiber extract as used in the dietary supplements in the high cereal fiber (HCF) and moderately high cereal fiber/protein (MIX) groups. BIC depicts the bicarbonate buffered medium used for solution of both glucose and the cereal fiber extract. SCFA, short chain fatty acids.

The here used cereal fiber extract contained mainly cellulose (70%) and hemicelluloses (30%), as described in detail [12,20]. There was no relevant *in vitro *fermentation of the cereal fiber extract after incubation for 24 h and 46 h, whereas glucose solution that was used as control was strongly fermented (Figure 1).

### Diet induced changes in fecal SCFA profiles

Diet-induced effects on SCFA profiles were investigated in fecal samples after 0, 6, and 18 weeks of dietary intervention. Analyses for SCFA were performed in duplicates. Baseline levels (week 0) of fecal SCFA showed no significant differences between groups (Table 1). Apart from a significant effect in the full model for valeric acid (p = 0.037) which was driven mainly by a significant increase of this SCFA in the HP group after 6 weeks [week 0 (baseline) vs week 6, p = 0.002; week 0 vs week 18, p = 0.056, see Tables 1 and 3] and comparable to the increase seen with isovaleric acid that was used as a biomarker for protein intake [12], there were no diet-induced differences between groups in other SCFA (p > 0.28 for all parameters, Table 3). Subgroup analyses for valeric acid showed significant differences after 6 weeks of dietary intervention both between HP as compared with control (p = 0.028), and between HP as compared with HCF (p = 0.01; Table 3).

**Table 3:** Markers of colonic fermentation, according to diet, after 6 and 18 weeks.

		**6 weeks**					**18 weeks**			
				
	**control**	**HCF**	**HP**	**MIX**	**p value**	**control**	**HCF**	**HP**	**MIX**	**p value^1^**
**Fecal SCFA (mM)**										
Acetate (C2)	34.4 ± 3.3	42.2 ± 6.1	37.4 ± 2.8	41.7 ± 5.4	0.75	37.2 ± 2.6	44.1 ± 5.7	40.4 ± 3.9	39.8 ± 5.3	0.79
Propionate (C3)	12.8 ± 1.8	13.1 ± 1.7	15.0 ± 1.6	15.4 ± 1.6	0.28	14.1 ± 1.4	16.5 ± 2.2	17.4 ± 2.3	13.7 ± 1.2	0.77
Butyrate (C4)	11.3 ± 2.1	15.1 ± 2.8	15.6 ± 1.7	14.8 ± 2.7	0.41	13.3 ± 1.8	13.8 ± 2.0	12.9 ± 1.4	12.4 ± 1.7	0.98
Valerate (C5)	2.2 ± 0.3	1.9 ± 0.3	3.1 ± 0.3	2.4 ± 0.3	0.037	2.9 ± 0.3	2.4 ± 0.3	3.1 ± 0.4	2.6 ± 0.3	0.48
Sum SCFA^2^	63.7 ± 6.4	74.8 ± 9.5	75.6 ± 5.9	77.6 ± 9.0	0.42	71.6 ± 5.6	80.4 ± 9.7	78.2 ± 7.2	72.1 ± 7.3	0.90
**Fecal dry mass (FDM, %)**										
FDM (%)	25.4 ± 2.1	34.3 ± 2.7	28.2 ± 1.9	28.3 ± 1.8	0.08	27.8 ± 1.4	30.4 ± 2.7	27.0 ± 1.7	30.1 ± 2.3	0.35

All values are mean ± SEM. Baseline values are shown in Table 1. HCF, diet high in cereal fiber; HP, diet high in protein; MIX, diet moderately high in both cereal and protein. ^1^P values refer to the respective treatment × time interaction in the full model. ^2^Sum SCFA includes the measurement of fecal iso-acids

Re-analysing all the here investigated markers after normalizing for baseline value levels (week 0) again did not show any significant diet induced effects on fecal SCFA. In addition, diet-induced effects on fecal valeric acid concentrations failed to reach significance level in the full model after correction for baseline levels (6 weeks, p = 0.076; 18 weeks, p = 0.90).

Diet induced differences within groups when comparing data longitudinally (week 0 vs week 6 and 18, respectively) are shown in Table 3.

### Breath hydrogen concentrations

Results from investigating hydrogen breath concentrations as a further marker for colonic carbohydrate fermentation have been published and were not different between dietary groups in all analyses [12].

### Effects on potential targets of diet-induced SCFA

In agreement with unchanged fecal SCFA profiles with the HCF diet there were no significant changes in commonly proposed factors that have been shown to be influenced by SCFA [1,16,30], which included circulating levels of the orexigenic gut hormone ghrelin (full model, 6-weeks, p = 0.81, 18-weeks p = 0.85), and markers of inflammation (hsCRP, 6-weeks, p = 0.33, 18-weeks p = 0.41; PAI-1, 6-weeks, p = 0.50, 18-weeks p = 0.61; and IL-10, 6-weeks, p = 0.46, 18-weeks p = 0.46).

### Diet-induced effects on fecal dry mass

Fecal dry mass significantly increased in the HCF group between 0 and 6 weeks (p = 0.009), but was comparable to baseline after 18 weeks (p = 0.56) (Table 3). Fecal dry mass also tended to increase in the MIX group after 18 weeks (p = 0.092), but not after 6 weeks (p = 0.22) of dietary intervention (Table 3). There were no diet-induced effects on fecal dry mass in the control (6 weeks, p = 0.71; 18 weeks, p = 0.35) and in the HP groups (6 weeks, p = 0.24; 18 weeks, p = 0.7) (Table 3).

### Diet-induced effects on whole-body insulin sensitivity

Results of diet-induced effects on whole-body insulin sensitivity in the entire study group have been published [12]. We here present results from sub-group analyses, including all participants that were not excluded according to the study protocol and provided sufficient fecal samples on all study days (n = 69). The isoenergetic dietary intervention significantly influenced whole-body insulin sensitivity (mixed-model analysis for repeated measures, treatment × time interaction, 0-18 weeks: p = 0.046; absolute values: M-value 3.8 ± 0.4 (week-0) vs 4.3 ± 0.4 mg·kg^-1^·min^-1 ^(week-6), p = 0.015; and M-value 3.8 ± 0.4 (week-0) vs 4.5 ± 0.6 mg·kg^-1^·min^-1 ^(week-18), p = 0.065). Diet induced effects on insulin sensitivity expressed as percentage of the baseline value after 6 and 18 weeks were comparable to the results in the entire study group (ANOVA, week-6, p = 0.034; week-18, p = 0.27). Therefore the subgroup described here showed similar changes in insulin sensitivity as the entire study group [12].

### Further outcomes

Effects of the here investigated isoenergetic diets on body fat distribution and liver fat content have been published and showed no differences between groups [8].

## Discussion

Colonic fermentation of undigested dietary fibers with the production of SCFA has been proposed as one of the potential mechanisms that may explain improved insulin sensitivity and reduced diabetes risk in subjects consuming high fiber diets including diets high in cereal fibers [1,3,4,31,32].

We here show that implementation over a period of 18 weeks of a supplemented diet high in cereal fibers (> 40 g/day) markedly and significantly improved whole-body insulin sensitivity in the full model, but no differences between dietary groups in various markers of colonic carbohydrate fermentation including fecal SCFA, breath hydrogen concentrations [8], and several of their proposed targets were detected. In subgroup analyses, the only detected diet-induced effect was an increase of fecal valerate concentrations in the HP group which was expected in agreement with the also observed increase in fecal isovalerate [12] that has been proposed as a biomarker for dietary protein intake [33].

We further investigated diet-induced effects on dominant groups of the gut microbiota that are known to be involved in carbohydrate fermentation and again found no significant changes in the here investigated microbiota groups. *Roseburia *tended to increase in the HCF and to decrease in the HP group which was in agreement with a previous study showing decreased *Roseburia *with a lower carbohydrate intake [27]. The dietary supplements of the HCF and MIX groups also showed no *in vitro *fermentation, and changes in hydrogen breath tests were comparable between groups, probably explained by the fact that all four diets including the control diet were based on plant rich foods.

Therefore, the HCF diet-induced effects on whole-body insulin sensitivity were not matched by changes in markers of colonic fermentation and/or the composition of the gut microbiota, neither in the full model nor in additionally performed uncorrected subgroup analyses, and there was also no tendency to more pronounced effects after 18 versus 6 weeks of dietary intervention. The magnitude of improvement of insulin sensitivity with the HCF diet was comparable to results obtained from previous short-term (24 hours and 72 hours) randomized controlled cross-over studies from our laboratory, using the same cereal fiber extract [11,20], and independent from the ability of various insoluble fibers to change markers of colonic fermentation [20]. Furthermore, effects of cereal fiber intake on markers of glucose metabolism have been shown as short as 75 min after its intake, an interval that is likely too short to induce relevant changes on the gut microbiota [2]. In the few available long term studies in rodents that investigated otherwise comparable high fiber diets differing in their fermentation rate the short term beneficial effects of highly fermentable dietary fibers were abolished after prolonged intake, resulting in increased body weight and higher insulin resistance [34,35]. This was likely explained by a contribution of higher SCFA production to total energy intake [34], an effect that has been suggested to be relevant also in humans [36]. Most importantly, data from large prospective cohort studies consistently indicate that a high cereal fiber intake reduces diabetes risk as much as 30%, whereas more readily fermentable sorts of dietary fibers from fruit and vegetables show inconclusive results on this outcome [7,8,37]. We therefore suggest that improved insulin sensitivity with cereal fibers may not depend on the concept of colonic carbohydrate fermentation.

Strengths of our study included the state-of-the-art measurement of insulin sensitivity, the provision of isoenergetic diets, the provision of supplements to enhance discrimination between diets, and the use of biomarkers to assess compliance [12]. Limitations were: an 18-weeks intervention might have been too short to detect changes in the gut microbiota and/or fecal SCFA [1], and other here not measured factors (such as bile acids [38]) might have been influenced by the HCF diet. Furthermore, when FISH is used only selected bacterial groups are targeted depending on the selected probes. With a probe set covering essentially the same bacterial groups, coverage of 80% of the total human microbiota was shown previously [27]; however, complete coverage would require a non-targeted 16S rRNA gene-sequencing approach [39,40] that has not been available in this study.

Finally, we investigated changes of SCFA in fecal samples and the fraction of SCFA that was potentially absorbed by the host was not measured; analysis of SCFA in portal blood samples would be desirable but is not feasible in dietary intervention studies in humans, whereas the measurement of SCFA in peripheral blood faces various limitations, due to interference with endogenous production of acetate and butyrate by fat oxidation, and changes in propionate due to amino acid metabolism [1]. Since we did not observe any HCF induced increase of SCFA at the anatomical site of production (fecal samples from the colon), it is unlikely that changes in peripheral (and/or portal) blood in the HCF group would have been detected, and if so, these were unlikely to be explained by HCF-induced increases in colonic fermentation since colonic/fecal SCFA were unchanged in this group.

Despite these limitations, the combination of results including lack of *in vitro *fermentation of the cereal fiber extract as used for preparation of the dietary supplements in the HCF and MIX groups, no diet-induced changes in breath hydrogen concentrations [12], no changes in the composition of dominant groups of the gut microbiota, and unchanged fecal SCFA concentrations including the measurement of several of their proposed targets (serum ghrelin, several inflammatory markers) did not support the hypothesis that cereal fiber induced production of SCFA was a key driver in the here observed rapid onset improvement of whole-body insulin sensitivity with the HCF diet.

## Conclusions

Potential changes in SCFA and dominant groups of the gut microbiota in longer-term interventions may further contribute to beneficial effects of HCF diets. However, our here provided data do not lend support to the hypothesis that colonic carbohydrate fermentation with the production of SCFA was one of the key mechanisms mediating the observed rapid onset and significant improvement of whole-body insulin sensitivity in overweight subjects consuming a supplemented HCF-diet.

## List of abbreviations

HCF: high cereal fiber; HP: high protein; SCFA: short chain fatty acids; BMI: body mass index; FISH: fluorescence in situ hybridization; S6K1: serine kinase 6-1.

## Competing interests

The authors declare that they have no competing interests.

## Authors' contributions

MOW and AFHP were responsible for the design of the study. MOW performed the statistical analyses and drafted the manuscript. MB and CA were responsible for the analyses of dominant groups in the microbiota and short chain fatty acids in fecal samples. NB was responsible for the analysis of in vitro fermentation of the cereal fiber extracts. VL contributed to the acquisition of data. AMA and MM contributed to the analyses and interpretation of the data. All authors read and approved the final manuscript.

## Supplementary Material

Additional file 1 **Table S1: **FISH analysis - probes. additional file 1 shows the probes used for the analysis of diet-induced changes in gut microbiota composition. Click here for file
