# Supplementary material for: Changes in dominant groups of the gut microbiota do not explain cereal-fiber induced improvement of whole-body insulin sensitivity
Source: Nutr Metab (Lond). 2011 Dec 17;8:90. doi: 10.1186/1743-7075-8-90 (PMC3264513; doi:10.1186/1743-7075-8-90)
Supplement: Additional file 1 — Table S1: FISH analysis - probes. additional file 1 shows the probes used for the analysis of diet-induced changes in gut microbiota composition. [file 1743-7075-8-90-S1.DOC]

**Additional file 1, Table S1:** Probes used for the analysis of diet-induced changes in gut microbiota composition

| Probe | Oligonucleotide sequence |
| --- | --- |
| EUB338-Cy5 | 5´-GCT GCC TCC CGT AGG AGT-3´ |
| nonEUB338-Cy5 | 5´-ACA TCC TAC GGG AGG C-3´ |
| nonEUB338-FITC | 5´-ACA TCC TAC GGG AGG C-3´ |
| Bac303-FITC | 5´-CCA ATG TGG GGG ACC TT-3´ |
| Erec482-FITC | 5´-GCT TCT TAG TCA RGT ACC G-3´ |
| Rrec584-FITC | 5´-TCA GAC TTG CCG YAC CGC-3´ |
| Lab158-FITC | 5´-GGT ATT AGC AYC TGT TTC CA-3´ |
| Clept866-FITC | 5´-GGT GGA TWA CTT ATT GTG-3´ |
| cp Clept1 | 5´-GGT GGA AWA CTT ATT GTC-3´ |
| cp Clept2 | 5´-GGT GGA TWA CTT ATT GTG-3´ |
| Ato291-FITC | 5´-GGT CGG TCT CTC AAC CC-3´ |
| Bif164-FITC | 5´-CAT CCG GCA TTA CCA CCC-3´ |
| Prop853-FITC | 5´-ATT GCG TTA ACT CCG GCA C-3´ |
| Enter1432-FITC | 5´-CTT TTG CAA CCC ACT-3´ |
